# Cofactor Engineering for Enhancing the Flux of Metabolic Pathways

**DOI:** 10.3389/fbioe.2014.00030

**Published:** 2014-08-28

**Authors:** M. Kalim Akhtar, Patrik R. Jones

**Affiliations:** ^1^Department of Biochemical Engineering, University College London, London, UK; ^2^Department of Life Sciences, Imperial College London, London, UK

**Keywords:** cofactors, metabolic pathway engineering, Fe–S clusters, enzymatic activity, synthetic biology

## Abstract

The manufacture of a diverse array of chemicals is now possible with biologically engineered strains, an approach that is greatly facilitated by the emergence of synthetic biology. This is principally achieved through pathway engineering in which enzyme activities are coordinated within a genetically amenable host to generate the product of interest. A great deal of attention is typically given to the quantitative levels of the enzymes with little regard to their overall qualitative states. This highly constrained approach fails to consider other factors that may be necessary for enzyme functionality. In particular, enzymes with physically bound cofactors, otherwise known as holoenzymes, require careful evaluation. Herein, we discuss the importance of cofactors for biocatalytic processes and show with empirical examples why the synthesis and integration of cofactors for the formation of holoenzymes warrant a great deal of attention within the context of pathway engineering.

## Introduction

Synthetic biology permits the engineering of biological devices or systems with novel or enhanced functions (Church et al., 2014). Such an approach has numerous applications, most notably in the manufacture of a diverse number of molecules including household chemicals, biofuels, and pharmaceutical drugs (Keasling, 2010). This is principally achieved through pathway engineering in which enzyme activities are carefully coordinated to generate the product of interest. Approaches based on the activities of isolated enzymes (*in vitro*) or whole cells (*in vivo*) can be employed for this purpose (Guterl et al., 2012; Stephanopoulos, 2012). The latter approach in particular offers a significant benefit with respect to complex, multi-step pathways that rely on secondary cellular factors, as well as additional pathway processing. Since the enzymes serve as the workhorse components of these biocatalytic systems, both their quantitative levels and qualitative states are important parameters to consider for pathway engineering. A great deal of focus is typically placed on the quantitative levels of the enzyme (Zelcbuch et al., 2013) with little attention given to their overall qualitative states. Thus, the assumption is usually made that these enzyme components are functioning at full capacity. However, this may not always be the case given that a large subset of enzymes depend on cofactors for functionality.

## Significance of Cofactors in Biology

All biological organisms possess a network of pathways that lead to the production of metabolites with an array of cellular functions (Feist et al., 2009). The synthesis and interconversion of these metabolites is made possible by the catalytic activities of countless enzymes. By lowering the activation energy barrier, enzymes catalyze reactions at considerably faster rates than their chemical counterparts. This characteristic property along with the relatively high degree of substrate selectivity, permit the use of enzymes as catalysts for industrial purposes. For those enzymes, which rely solely on amino acids for catalysis, the types of reactions are extremely narrow in scope and, for the most part, restricted to acid/base and electrophilic/nucleophilic reactions (Broderick, 2001). To further extend, the range of reactions, enzymes are commonly associated with non-protein moieties known as cofactors (Broderick, 2001).

In the broadest sense of the term, cofactors are thought to be associated with well over half of known proteins (Fischer et al., 2010a). However, for this article, the term “cofactor” will refer specifically to those moieties, either organic or inorganic, which remain physically associated with the enzyme throughout the catalytic cycle (Fischer et al., 2010a). This excludes dissociable cosubstrates such as NADPH and glutathione. Additionally, since the primary focus of this article is on pathway engineering, only those cofactors that require *de novo* pathways for syntheses will be mentioned and sole metal entities such as calcium and selenium will also be excluded. Using this strict definition, a selection of common cofactors are listed in Table 1. These are categorized into two types: organic and inorganic (Rees, 2002; Fischer et al., 2010b). Members of the organic group of cofactors tend to be derivatives of vitamins and undertake numerous types of reactions, while the inorganic group is usually based on various arrangements of iron–sulfur (Fe–S) clusters.

**Table 1:** **Examples of enzyme bound cofactors**.

Enzyme bound cofactor	Type of reaction catalyzed	Example of a cofactor-containing enzyme	Associated pathway
**ORGANIC COFACTORS**			
Biotin	Carbon dioxide addition	Acetyl CoA carboxylase	Fatty acid biosynthesis
Factor F430	Methyl transfer	Methyl coenzyme M reductase	Methanogenesis
Flavin mononucleotide	Electron transfer	Cytochrome P450 reductase	Detoxification
Heme	Electron transfer	Cytochrome P450	Detoxification
Lipoic acid	Acyl/methyl amine transfer	2-oxoacid dehydrogenase	Citric acid cycle
MIO cofactor	Carbon–hydrogen bond activation	Phenylalanine ammonia-lyase	Polyphenol biosynthesis
Molybdopterin	Electron transfer	Xanthine oxidase	Purine catabolism
Phosphopantetheine	Acyl carrier	Carboxylic acid reductase	Fatty acid metabolism
Pyridoxal 5′phosphate	Transamination	Glycogen phosphorylase	Glycogenosis
Pyrroloquinoline quinone	Electron transfer	Methanol dehydrogenase	Methane metabolism
Thiamine pyrophosphate	Carbon dioxide removal	Pyruvate ferredoxin/flavodoxin reductase	Pyruvate decarboxylation
Topaquinone	Amine oxidation	Amine oxidase	Urea cycle
**INORGANIC COFACTORS**			
Fe–S	Electron transfer	Ferredoxin	Iron–sulfur cluster biogenesis
H-cluster	Hydrogen activation	Fe–Fe hydrogenase	Hydrogen metabolism
Fe-Moco	Nitrogen reduction	Nitrogenase	Nitrogen fixation
C-cluster	Carbon monoxide oxidation	Carbon monoxide dehydrogenase	Carbon monoxide metabolism
P-cluster	Electron transfer	Nitrogenase	Nitrogen fixation

*For a more comprehensive list of organic cofactors refer Fischer et al. (2010b)*.

**Figure 1:**
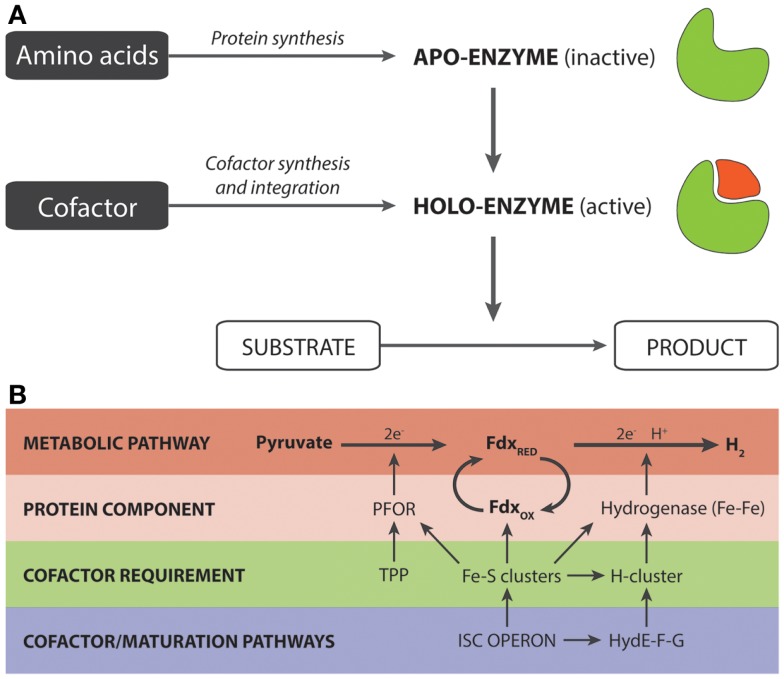
**(A)** Generalized overview of the synthesis of holoenzymes. **(B)** Significance of cofactor engineering for enhancing the output of holoenzyme-dependent pathways. The example (Akhtar and Jones, 2009) illustrates a synthetic pyruvate:H2-pathway that is heavily dependent on Fe–S clusters. These clusters are required for (i) the proteins/enzymes directly involved in the pathway for hydrogen production and (ii) the maturation factors that are responsible for the synthesis and integration of the H-cluster present in Fe–Fe hydrogenases.

In its cofactor-bound state, enzymes are referred to as holoenzymes while in the unbound state, they are known as apoenzymes (Figure 1A). Two discrete structural parts are required for the synthesis of a holoenzyme: a polypeptide chain and a cofactor moiety. The former is generated by the ubiquitous translational machinery while the latter, depending upon the cofactor, is synthesized by a defined metabolic pathway. In certain cases, subtle variations of the cofactor pathway may exist. For example, in animals, fungi, and α-proteobacteria, heme synthesis is initiated by 5-aminolevulinate synthase, via condensation of glycine and succinyl CoA, while in photosynthetic eukaryotes and some species of the α-proteobacterial group, it depends on glutamate, via the concerted actions of three enzymes (Layer et al., 2010). Once synthesized, the cofactor is integrated with the apoenzyme, either in a co-translational or post-translational manner, to form the holoenzyme. The nature of the association may be covalent and, in such cases, linkages are typically formed with serine, threonine, histidine, tyrosine, and lysine residues and catalyzed by a distinct maturation system. As an example, the heme *c* in cytochrome *c* is attached to a cysteine residue, via the vinyl group with the aid of Ccm (cytochrome *c* maturation) factors (Sanders et al., 2010). Alternatively, the interaction may be tight but non-covalent as in the case of the flavin-containing enzyme, acyl CoA dehydrogenase (Thorpe and Kim, 1995).

## Importance of Cofactor Synthesis for Both Enzyme and Pathway Functionality

As integral components of numerous holoenzymes, cofactors are required for the majority of metabolic pathways. To name but a few, these include: FAD in succinate dehydrogenase for the Krebs cycle; TPP in transketolase for the pentose phosphate pathway; pyridoxal phosphate in glycogen phosphorylase for glycogenolysis; H-clusters in hydrogenases for hydrogen metabolism; Fe–MoCo in nitrogenases for nitrogen fixation; biotin in acetyl CoA carboxylase for fatty acid biosynthesis; and haem in cytochrome P450 for various detoxification pathways. The most crucial point to consider is that the functional output of holoenzymes can only arise if the apoenzyme is correctly folded with its cofactor. Without the cofactor, such enzymes would be rendered inoperable and the associated pathways would become redundant. This situation, though undesirable, is likely to be encountered in a bottom-up approach to microbial engineering in which a genetically amenable and well-characterized organism, such as *Escherichia coli* and *Saccharomyces cerevisiae* may be completely devoid of cofactors necessary for heterologous enzyme activity. Alternatively, the capability of the host to synthesize the required cofactor exists but may be insufficient, resulting in a pool of enzymes with a high ratio of apo to holo form (see next section). The most obvious strategy for resolving these issues would be to resort to “cofactor engineering,” by genetically modifying the host so that the cofactor assembly system is present or that the level of native cofactor assembly is in sufficient supply.

Consider the expression of the clostridial Fe–Fe hydrogenase. This enzyme depends on an H-cluster that essentially is a di-iron arrangement with three carbon monoxide, two cyanide ligands, and one dithiolate bridge (Mulder et al., 2011). The formation of this cluster is catalyzed by three maturation enzymes; namely HydE, HydF, and HydG (Posewitz et al., 2004). Since *E. coli* is not naturally endowed with the Hyd maturation enzymes, *E. coli* invariably produces a non-functional Fe–Fe hydrogenase. This can be circumvented by simply complementing the expression of Fe–Fe hydrogenases with the maturation pathway for the H-cluster in order to form the active Fe–Fe hydrogenase (Posewitz et al., 2004; Akhtar and Jones, 2008b). Pyrroloquinoline (PQQ) is another example of a cofactor, which is not naturally synthesized in *E. coli* (Matsushita et al., 1997). This cofactor, present in a family of quinoproteins, has potential uses in biofuel cells, bioremediation, and biosensing (Matsushita et al., 2002). By incorporating the *pqqABCDE* gene cluster from *Gluconobacter oxydansa*, Yang et al. (2010) were able to successfully demonstrate the activity of a PQQ-requiring d-glucose dehydrogenase in *E. coli*. They noted, however, that the gene cluster was most likely complemented by the native *tldD* gene. A final example is tetrahydrobiopterin, which in itself is a desirable commodity for the treatment of mild and moderate forms of phenylketonuria (Perez-Duenas et al., 2004). It can be synthesized *in vivo* from GTP via a three-step pathway comprising GTP cyclohydrolase I, 6-pyruvoyl-tetrahydropterin synthase, and sepiapterin reductase (Yamamoto et al., 2003). By augmenting the pathway with expression of a GTP cyclohydrolase I sourced from *Bacillus subtilis*, a 1.5-fold improvement was observed with titers reaching as high as 4 g biopterin per liter of culture (Yamamoto et al., 2003).

## Cofactor Insertion is Key to Maximizing Total and Specific Holoenzyme Activity

To maximize the specific activity of a holoenzyme, cofactor synthesis would need to be complemented and/or coupled with cofactor insertion. For enzymes, which bind to cofactors in a non-covalent fashion, the process of cofactor insertion is somewhat of an enigma. It is still not known, even for the well-known heme *b* cofactor, whether this process is facilitated by dedicated *in vivo* components or is a spontaneous process (Thöny-Meyer, 2009). Though overwhelming *in vitro* data show that heme *b* insertion can be a spontaneous event, recent evidence with *in vivo* model systems have implicated the involvement of cellular factors that have yet to be elucidated (Waheed et al., 2010; Correia et al., 2011). For those enzymes that have covalently attached cofactors, specialized maturation systems have evolved to catalyze both insertion and covalent linkage of the cofactor. Induction of the maturation system can increase holoenzyme activity, as in the case of a carboxylic acid reductase (CAR), which was recently employed for the production of a broad range of chemical commodities (Akhtar et al., 2013). Venkitasubramanian et al. (2007) had verified that CAR requires a cofactor known as phosphopantetheine. This cofactor, during its synthesis, is concomitantly integrated with the enzyme, via a phosphodiester bond, by a maturation enzyme known as phosphopantetheinyl transferase. The sole expression of CAR in *E. coli* leads to an observable, but exceedingly poor, activity. However, with coexpression of the phosphopantetheinyl transferase Sfp from *Bacillus subtilis*, the specific activity of CAR can be enhanced several-fold to a level that is on par with one that is purified from the native organism.

In addition to stimulating the specific enzyme activity, increasing the intracellular levels of cofactors is also known to improve the overall production levels of holoenzymes, suggesting a relationship between holo/apo-forms and degradation. This is a phenomenon that has been frequently observed for hemoproteins (Harnastai et al., 2006; Lu et al., 2010, 2013; Michener et al., 2012). By elevating heme levels, via supplementation of the media with δ-aminolevulinic acid, the expression levels of hemoglobin and cytochrome *b*_5_ can be significantly improved (Gallagher et al., 1992; Liu et al., 2014). Likewise, increasing Fe–S levels, via overexpression of the *isc* (Fe–S cluster) operon, also leads to similar effects (Nakamura et al., 1999; Akhtar and Jones, 2008a). An explanation for the increased holoenzyme levels may be gleaned from studies of proteins, which utilize divalent metal ions as cofactors (Wilson et al., 2004; Bushmarina et al., 2006; Palm-Espling et al., 2012). Evidence from these published reports suggests that cofactors may aid in the folding of the polypeptide chain and, in turn, accelerate the formation of a functional protein (Goedken et al., 2000). In the case of ribonuclease HI, metal cofactor integration was found to impart a greater degree of rigidity on the final native conformational state of the protein, in addition to improving the refolding rate of the protein (Wittung-Stafshede, 2002). Further insights on the importance of cofactors in protein folding can be gained with the *S*-adenosylmethionine-containing biotin synthase. This enzyme relies on an intact Fe–S cluster for the addition of sulfur to dethiobiotin to form the biotin thiophane ring. Reyda et al. (2008) noticed that the loss of the Fe–S cluster destabilized the protein, which led to transient unfolding of specific regions, as well as increased proteolysis. Proteolytic degradation was found to proceed by an apparent ATP-dependent proteolysis mechanism, via sequential cleavage of small C-terminal fragments. Interestingly, it was also speculated that since the activity of the protein is generally well maintained under high-iron conditions, a repair process, possibly mediated by the Isc and/or Suf (Sulfur mobilization) machinery, may be active under conditions of destabilization (Reyda et al., 2008).

## Stimulating Cofactor Synthesis Can Enhance the Flux of Synthetic Pathways

With regard to the actual impact that cofactor engineering can have on the metabolic performance of synthetic pathways, two studies are particularly worthy of mention. In the first study relating to the production of the vitamin C precursor, 2-keto-l-gulonic acid, Gao et al. (2013) utilized two PQQ-dependent dehydrogenases with d-sorbitol as the starting substrate. The authors noted that induced expression did not improve titers beyond a certain threshold and hypothesized that PQQ was the limiting factor. This was proven to be correct since induction of a pathway for PQQ synthesis resulted in a 20% increase in overall titer. A later refinement of the work in which the two pathway enzymes were incorporated as a fusion protein in *Ketogulonigenium vulgare* also resulted in a similar improvement in titer (Gao et al., 2014).

The second study relates to a synthetic pathway for hydrogen production consisting of a pyruvate:ferredoxin oxidoreductase (PFOR, also known as YdbK in the literature), Fdx and Fe–Fe hydrogenase (Akhtar and Jones, 2009). The design of this pathway was based on the observation that pyruvate, rather than NAD(P)H, was a metabolically superior source of electrons for hydrogen synthesis (Veit et al., 2008). Initial structural analysis of the PFOR-based pathway, in addition to the maturation enzymes, revealed that each protein component was associated with at least one Fe–S cluster that essentially provides the route of electron transfer from pyruvate to the H-cluster of the Fe–Fe hydrogenase (Figure 1B). Up to a total of 12 Fe–S clusters were found to be required for the pathway. In *E. coli*, the formation of Fe–S clusters is undertaken by the *isc* operon, though an analogous *suf* operon is also present (Fontecave et al., 2005; Roche et al., 2013). Since the *isc* operon is controlled by the negative IscR transcriptional regulator, our initial work using a Δ*iscR* background strain had shown that the levels of holo-ferredoxin and the *in vitro* hydrogenase activity could be improved two and threefold, respectively, relative to the wild-type strain (Akhtar and Jones, 2008a). Based on this insight and given the heavy demand for Fe–S clusters, we reasoned that the Δ*iscR* strain may improve the pathway flux toward hydrogen production by increasing the availability of Fe–S clusters. In accordance with our prediction, we observed a twofold improvement in hydrogen yield relative to the wild-type, resulting in an overall yield of 1.5 moles of H_2_ per mole of glucose. Even more remarkably with addition of TPP, which serves as a cofactor for the PFOR component, the hydrogen yield was further increased to give a final yield of 1.9, out of a theoretical yield of two moles per mole of glucose (Akhtar and Jones, 2009). Given also that both the specific and total *in vitro* activity of PFOR was improved, this suggests that the availability of the TPP cofactor may well be another potential limiting cofactor for hydrogen production (Akhtar and Jones, 2009).

Although cofactor biosynthesis and integration under native control can be limiting and unresponsive to high-expression levels of the apoform, presumably to conserve cellular resources, cofactor engineering can quite clearly be advantageous for the assembly of metabolic pathways that depend on the activity of heterologous holoenzymes. This benefit presumably arises from the improved holoenzyme activity, via increased structural stability and/or folding rates in conjunction with reduced protein degradation and/or protein unfolding (explained in the previous section). Interestingly, data from our work on the *in vitro* activity of Fe–S proteins also suggests that, under certain conditions, a steady and constant supply of cofactors may well aid the restoration of damaged or inactivated enzymes (Akhtar and Jones, 2008a).

## Concluding Remarks

Achieving a balanced production of polypeptide and cofactor for optimal holoenzyme activity would be an iterative process involving the (i) modulated induction of genes, (ii) monitoring of cofactor levels, (iii) evaluation of enzyme activity, and (iv) evaluation of whole-system productivity. Genetic modulation can be controlled at the transcriptional and translational levels by varying the strengths of promoter and ribosomal binding sites, while cofactor levels can be monitored and quantified using suitable analytical methods, e.g., mass spectrometry, high-performance liquid chromatography (HPLC). Combining this with information relating to specific enzyme activity should allow provide profound insights into the intracellular cofactor levels required to achieve optimal holoenzyme activity. Furthermore, if a product reporter system is available, it may also be possible to screen for optimal cofactor metabolism, for example using RBS-variation libraries (Zelcbuch et al., 2013).

Given the importance of cofactor synthesis and integration for holoenzyme activity, a few key points need to be considered when assembling pathways involving holoenzymes. Firstly, holoenzyme activity will only be possible within a host that is metabolically equipped to synthesize the necessary cofactor, otherwise a complementary pathway for cofactor production would also need to be implemented. This is particularly relevant for synthetic pathways that employ holoenzymes from diverse origins. Secondly, to ensure maximal activity of the holoenzyme, the apoenzyme needs to be sufficiently coupled with the synthesis and insertion of its respective cofactor. An imbalance between the two will lead to poor enzyme activity, one that would most likely be inadequate for catalytic purposes. Even native cofactor biosynthesis may not be optimally tuned or responsive to demand from an over-expressed apoenzyme, as would be required in a biocatalytic system where the objective function has shifted from biomass to metabolite producer. Thirdly, since cofactor stimulation is known to improve the stability and activity of holoenzymes, cofactor engineering is likely to be a useful strategy for enhancing the total activity of all holoenzymes in the engineered pathway, and maximizing chances for high flux toward the product of interest. As it currently stands, the impact of cofactor engineering on the activity of holoenzymes is still very much a poorly studied area and certainly warrants more attention given its potential impact on the success of engineered biocatalytic systems.

## Conflict of Interest Statement

The authors declare that the research was conducted in the absence of any commercial or financial relationships that could be construed as a potential conflict of interest.
